# Electroacupuncture and Brain Protection against Cerebral Ischemia: Specific Effects of Acupoints

**DOI:** 10.1155/2013/804397

**Published:** 2013-05-12

**Authors:** Fei Zhou, Jingchun Guo, Jieshi Cheng, Gencheng Wu, Jian Sun, Ying Xia

**Affiliations:** ^1^Shanghai Research Center for Acupuncture and Meridians, Shanghai 201203, China; ^2^Gongli Hospital, Pudong New District, Shanghai 200135, China; ^3^Shanghai Medical College of Fudan University, Shanghai 200032, China; ^4^The University of Texas Medical School at Houston, Houston, TX 77030, USA; ^5^Yale University School of Medicine, New Haven, CT 06520, USA

## Abstract

Electroacupuncture (EA) has been shown to increase cerebral blood flow (CBF) and reduce ischemic infarction in the rat model of cerebral ischemia (middle cerebral artery occlusion, MCAO). Since multiple acupoints are recommended to treat cerebral ischemia, we performed this study to investigate if there is any variation in EA protection against cerebral ischemia with the stimulation of certain “acupoints” in rats. One hour of right MCAO with an 85% reduction of blood flow induced an extensive infarction (32.9% ± 3.8% of the brain), serious neurological deficits (scale = 6.0 ± 0.5, on a scale of 0–7), and a 17% (10 out of 60) mortality. EA, with a sparse-dense wave (5 Hz/20 Hz) at 1.0 mA for 30 minutes, at Du 20 and Du 26 greatly reduced the infarction to 4.5% ± 1.5% (*P* < 0.01), significantly improved neurological deficit (scale = 1.0 ± 0.5, *P* < 0.01), and decreased the death rate to 7% (2 out of 30, *P* < 0.01). Similarly, EA at left LI 11 & PC 6 reduced the infarct volume to 8.6% ± 3.8% (*P* < 0.01), improved the neurological deficit (scale = 2.0 ± 1.0, *P* < 0.01), and decreased the death rate to 8% (2 out of 24, *P* < 0.01). In sharp contrast, EA at right LI 11 & PC 6 did not lead to any significant changes in the infarct volume (33.4% ± 6.3%), neurological deficit (scale = 6.5 ± 0.5), and the death rate (20%, 5 out of 24). EA at left GB 34 & SP 6, also had an inconspicuous effect on the ischemic injury. EA at Du 20 & Du 26 or at left LI 11 & PC 6 instantaneously induced a significant increase in cerebral blood flow. Neither EA at right LI 11 & PC 6 nor at GB 34 & SP 6 increased cerebral blood flow. These results revealed that the EA protection against cerebral ischemia is relatively acupoint specific.

## 1. Introduction 

Brain hypoxia/ischemia, as in stroke, causes neuronal injury [1–4] and results in neurological disability and death. Prevention and early treatment are paramount in reducing its devastating effects on affected individuals and their families. However, treatment strategies are presently very limited in spite of extensive research over several decades. Seeking new and effective therapy, including complementary and alternative approaches to prevent/treat ischemic injury, is of utmost importance.

Traditional Chinese Medicine (TCM) has long advocated the use of acupuncture to treat stroke and other neurological disorders. Indeed, several ancient TCM books, for example, Huangdi Nei Jing (*Huangdi's Internal Classic*)* and *“Handbook of Prescriptions for Emergencies” (*Zhou Hou Fang*), both contain descriptions of acupuncture therapy for stroke-like disorders. In recent years, a substantial amount of the literature has appeared on the use of manual acupuncture or EA for the brain protection against ischemic injury in both human subjects and animal models [5–15]. However, there are still controversies in terms of the clinical outcome [16–18]. The evidence on the effectiveness of acupuncture in stroke patients was inconclusive under clinical settings, which could be attributed to poor methodological quality and small samples. Further high-quality, randomized controlled trials with long-term followups are needed [18].

Acupuncture induces a complex effect on the central nervous system [19–22] and thus displays therapeutic effects on the body, which is influenced by multiple factors, especially stimulation manner/parameters and location (acupoints). Differences in various methods and acupoints of acupuncture manipulation may greatly affect immediate efficacy and long-term prognosis of patients. However, there is a lack of knowledge on optimal acupuncture conditions for the treatment of ischemic injury. Traditional Chinese Medicine recommends various acupoints located over different parts of the body along with different methods of acupunctural stimulation. However, most of these recommendations are based on personal experiences and have not been subjected to scientific testing. Therefore, optimizing acupuncture conditions for maximal efficacy is of critical value because different manipulations of acupuncture can result in completely different outcomes. For obvious reasons, it is difficult, if not impossible, to conduct such tests on patients and therefore must be done largely via animal studies.

Recently, we determined the appropriate EA parameters for cerebral protection against ischemia [9, 13]. In fact, our findings suggest that the current frequency and intensity for EA stimulation differentially alters cerebral blood flow and infarct volume in the rat brain exposed to middle cerebral artery occlusion (MCAO). Since there are multiple acupoints all over the body and EA effects are generally acupoint specific, it is equally important to define the specific roles corresponding to each relevant acupoint. Although there are sporadic studies that suggest that Baihui (Du 20) might be better than other acupoints for EA-induced protection against cerebral ischemia [11, 15, 23], a comparative evaluation of various acupoints has yet to be performed. 

In order to clarify the fundamental issue concerning the specificity of acupoints in EA-induced cerebral protection against ischemia, we performed a number of experiments on rats to investigate the specific outcomes of stimulation of acupoints located in various regions of the body under ischemic conditions. We specifically chose 4 pairs of “acupoints” in the head, left posterior limb, and left and right anterior limbs, respectively, in addition to the controls. We performed constant monitoring to record the effects on blood flow, neurological deficits, and ischemic infarction in the ischemic brain for a reliable comparison between all groups. Our present data, consistent with our preliminary findings [10], shows that the acupoints in the head and left (contralateral) anterior limb confer maximum protection against ischemic injury in the MCAO rat model, suggesting the importance of these acupoints in the cerebral protection in stroke.

## 2. Materials and Methods

### 2.1. Animals

Adult male Sprague-Dawley rats (250 g ± 10 g) were purchased from the Experimental Animal Center for Shanghai Chinese Academy of Science and housed at an ambient temperature of 24 ± 1°C with free access to food and water. All surgical procedures were performed under anesthesia (chlorate hydrate, 400 mg/kg, i.p.). All animal procedures were approved by the Animals Care and Use Committees of Fudan University Shanghai Medical College, Shanghai, China. 

### 2.2. Monitoring Cerebral Blood Flow

A laser-Doppler perfusion monitor (LDPM, PeriFlux5000, Perimed, Sweden) was used for the measurement of cerebral blood flow. The main procedure [24] was as follows. A little round hole located at 1.5 mm posterior to the bregma and 5 mm lateral to the sagittal suture was drilled in the right parietal bone and a laser Doppler probe (0.45 mm diameter) was then inserted at a depth of 2 mm to approach the superficial microvessels in the cerebral pia mater to measure blood perfusion of the cortex as an index of cerebral blood flow. The probe was then fixed on the skull bone. Changes in cerebral blood flow were continuously monitored in all animals beginning at least 5 min before the induction of cerebral ischemia to about 15 min after reperfusion (until animal revival). According to the manufacturer, change in perfusion values depends on the number of blood cells present in the area illuminated by the probe tip and the speed at which they move. The changes in blood perfusion recorded as a measure of the dynamic changes in the cerebral blood flow in a localized area were constantly monitored in real time using LDPM. The values measured by LDPM were automatically expressed as perfusion units (PUs), concentration of the moving blood cells (CMBC), and the velocity of blood cells (Velocity). Of these values, PU is the most important index of cerebral blood flow and represents the product of the relative number of moving blood cells and their relative velocities.

### 2.3. Induction of Cerebral Ischemia

The focal cerebral ischemia model was established by performing the middle cerebral artery occlusion (MCAO) based on the methods described by Longa et al. [25]. Briefly, the rats were surgically operated under anesthesia to expose their right common carotid, the external carotid, and the internal carotid arteries. After ligating the distal end of the right external carotid artery, it was incised and a segment of 4-0 monofilament nylon suture (30 mm length, 0.18 mm diameter with a 0.24 mm diameter round tip, MONOSOF, SN-1699G, USA) was inserted from the right external carotid artery into the right internal carotid artery for a length of ~20 mm (reaching the origin of the right middle cerebral artery) to occlude the blood flow of the right middle cerebral artery. 

To standardize our experimental conditions across all experimental groups, we constantly monitored the blood flow in all animals to make sure that a relatively uniform level of cerebral ischemia existed before experimentation. The blood flow was controlled by adjusting the suture in the artery for the induction of ischemia. The ischemic rats that showed a stable drop of ~85% in PU compared to the baseline level (before MCAO), that is, reaching a level of ~15% of the baseline PU, were used for further experimentation. This ischemic condition was kept constant with minor fluctuations during the entire ischemic period. After the occlusion, reperfusion of the ischemic area was allowed by withdrawing the suture from the right external carotid artery. Changes in cortical blood flow were constantly monitored in all animals beginning at least 5 mins before MCAO and continuing up to 15 mins after reperfusion (until animal recovered from the anesthesia). This period included the entire course of MCAO as well as the full length of EA.

Body temperature was monitored by a rectal thermometer and maintained at 36.5°C ± 0.5°C from the start of the surgical procedures till the animal recovered from anesthesia. After the reperfusion, the animals were housed for 24 hours at an ambient temperature of 24 ± 1°C. In the sham-operation group, the same surgical procedures other than artery occlusion were performed. 

### 2.4. Application of Electroacupuncture

EA was delivered to 4 pairs of “acupoints” to compare their effects on brain protection and blood flow, those being “Shuigou” (Du 26) and “Baihui” (Du 20), left “Quchi (LI 11)” and “Neiguan” (PC 6), right “Quchi (LI 11)” and “Neiguan” (PC 6), and left “Yanglingquan” (GB 34) and “Sanyinjiao (SP 6). As described previously [13, 19, 26], “Shuigou (Du 26)” and “Baihui (Du 20)” are acupoints on head and face. Shuigou (Du 26) is located in the center of the upper lip situated at a point 2/3rds from the mouth on a line connecting mouth and nose. Baihui (Du 20) is located on the midline of the head, approximately at the midpoint of the line connecting the apices of the two auricles. The depth of the needle used on Du 26 was 1 mm vertically into the skin and 2 mm obliquely into Du 20. “Quchi (LI 11)” and “Neiguan” (PC 6) are acupoints on the anterior limbs. “Quchi (LI 11)” is in the depression on the lateral side of the elbow joint, and “Neiguan” (PC 6) is at the suture between ulnar and radial bones with a distance of 3 mm superior to the wrist joint. Left “Quchi (LI 11)” and “Neiguan” (PC 6) are contralateral to the ischemic cerebral hemisphere. Right “Quchi (LI 11)” and “Neiguan” (PC 6) are ipsilateral to the ischemic cerebral hemisphere. The depth of the needle on LI 11 is 4 mm vertically to the skin and PC 6 is 2 mm vertically. “Yanglingquan” (GB 34) and “Sanyinjiao (SP 6) are acupoints on the posterior limbs. “Yanglingquan” (GB 34) is located in the depression below the capitulum fibulae posterolateral to the knee joint, and “Sanyinjiao (SP 6) is at a point 10 mm superior to the tip of the medial malleolus. Left “Yanglingquan” (GB 34) and “Sanyinjiao (SP 6) are contralateral to the ischemic cerebral hemisphere. The depth of the needle under GB 34 was 5 mm vertically to the skin and that under SP 6 was 6 mm vertically to the skin.

 The “optimal” stimulation parameters, that is, sparse-dense wave (5 Hz/20 Hz) at 1.0 mA, were chosen based on our previously described reports [9, 13]. In all four EA groups, the same parameters were adopted with only a difference in the acupoint stimulated. EA was started 5 min after the onset of MCAO and was continued for 30 min. It was delivered through stainless steel filiform needles (15 mm length with 0.3 mm in diameter, Suzhou Medical Apparatus Limit Co., China) by an EA apparatus (Model G-6805-II, Shanghai Medical Instruments High-Tech Co., China). This EA apparatus has been widely used in the practice of clinical acupuncture in Traditional Chinese Medicine. The intensity and frequency of the output waves with negative-going pulse on the posterior border (pulse width = 0.5 ± 0.1 ms; component of direct current = 0) were monitored by a general oscillograph (Model XJ4210A, Shanghai XinJian Instrument and Equipment Co., China). 

The ischemic rats with MCAO were randomly divided into 5 groups: MACO only (Ischemia, *n* = 60), Ischemia + EA at Du 20 and Du 26 (*n* = 30), Ischemia + EA at left LI 11 and PC 6 (*n* = 24), Ischemia + EA at right LI 11 and PC 6 (*n* = 24), and Ischemia + EA at left SP 6 and GB 34 (*n* = 24). Finally, to achieve a strict control, we applied comparable acupoint stimulation using the same EA parameters for both ischemic and nonischemic rats and measured the changes in their cerebral blood flow (*n* = 8) to determine if EA has an acupoint-specific effect on the cerebral blood flow under ischemia. 

### 2.5. Evaluation of Death Rate and Neurological Deficits

In our experiments, some ischemic rats died between 2 and 20 hrs after reperfusion. The death rates were calculated based on the number of dead rats within this period and the total number of rats in the given group. Although the deaths could be attributed to a myriad of causes including hemorrhage, we did not investigate the cause of death as part of the study objectives as our aim here was to compare the death rates among various groups. 

Neurological behaviors were evaluated in all groups, excluding the rats that died within 24 h after MCAO. Neurological deficits were evaluated at 24 h after the reperfusion (right before sampling brain tissue for detection of ischemic infarction and other tests). The evaluation of neurological deficits was blinded, that is, the one who judged and scored the degree of neurological deficits according to the preset criteria did so without any knowledge about the grouping and treatments. The degree of neurological deficits was graded on a scale of 0 to 7 [13]. The criteria were set as follows: Grade 0— “normal”, symmetrical movement without any abnormal signs; Grade 1—incomplete stretch of the left anterior limb when the tail was lifted up; Grade 2—doddery crawl along with the signs of Grade 1; Grade 3—kept the left anterior limb close to the breast when the tail was lifted up; Grade 4—left turn when crawling; Grade 5—left anterior claw pushed backward along with the signs of Grade 4; Grade 6—repeated rotational motion with an immotile posterior left limb; Grade 7—left recumbent position because of body supporting incapability.

### 2.6. Measurement of Cerebral Infarct Volume

Following the evaluation of neurological deficits at 24 h after reperfusion, the rats were sacrificed under anesthesia, and sections of their brains were obtained as 2.0 mm slices (*n* = 12 ~ 18). The brain slices were incubated in a solution of triphenyltetrazolium chloride (TTC, 20 g/L) for 30 minutes at 37°C and then transferred into paraformaldehyde solution (40 g/L) to fix the area of infarct. The infarct region presented as white or pale in color while “normal” tissue showed up as red [13, 27, 28]. The images of the brain slices were taken with a digital camera attached to a computer system. The area/volume of infarct was analyzed by a computer-assisted image system with ACT-2U software (Nikon). Relative infarct ratio was calculated using the following equation [24]: (2 ∗ left hemisphere area (non-ischemic side) −noninfarct area of whole brain slices)/(2 ∗ left hemisphere areas) ∗ 100%. This equation excludes the factors that could result in an inaccurate calculation of the infarct volume (such as edema). 

### 2.7. Data Analysis

Cerebral blood flow was determined as a measure of PU, CMBC, and Velocity. All values in each animal were compared to the baseline values measured before MCAO. The grouped values were compared across various groups. Neurological deficits were evaluated and expressed as an average value of the grade per group. Cerebral infarct volume was expressed as a percentage of the whole cerebrum. 

All data are presented as mean ± SD and subjected to statistical analysis. The rate of death was compared using the Chi-square test. All other data were subjected to Analysis of variance (ANOVA), *t*-test, Rank-Sum test, and/or Chi-square test. The changes were considered as significant if the *P* value was less than 0.05. 

## 3. Results

### 3.1. EA Protection against Ischemic Injury Varied with Different Acupoints

**Figure 1:**
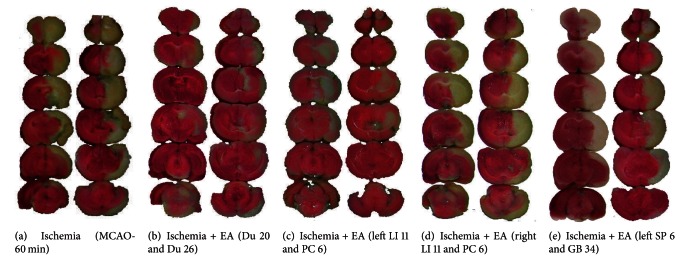
EA-induced reduction in the infarct volume varied with stimulation of different acupoints. The brain slices were subjected to TTC staining and the ischemic infarct volume was quantified by a computerized image system. Note that the infarct region (pale-white portion) was mainly located in the striatum and the frontoparietal cortex in the right hemisphere. The slices on the *right *of each column show the backside of the *left *slices. The MCAO-induced infarction (a) was significantly reduced by EA treatment at the acupoints Du 20 and Du 26 (b) and LI 11 and PC 6 on the left anterior limb (c). In contrast, EA treatment with the same parameters at the acupoints LI 11 and PC 6 on the right anterior (d) and SP 6 and GB 34 on the left posterior limbs (e) enabled no appreciable protection against cerebral infarction.

 In the Ischemia group (MCAO only, *n* = 60), 17% (10/60) of the rats died in less than 24 hrs after 60 min of MCAO. At 24 h after reperfusion, the remaining rats showed serious neurological deficits (Grade 6.0 ± 0.5, *n* = 50) with a cerebral infarct volume around one-third of the volume of the whole brain (32.9%  ± 3.8%, *n* = 18) (Table 1, and Figure 1(a)). The infarct areas were extensively distributed in the right frontoparietal lobe of the cortex and the striatum. The ischemic side was very swollen. 

In the group of Ischemia + EA at Du 20 and Du 26 (*n* = 30), 7% (2/30) of rats died within 2–10 hrs after MCAO (*P* < 0.01 versus Ischemia). At 24 h after reperfusion, the remaining rats showed a significant improvement of neurological deficits as compared to the ischemia alone group (Grade 1.0 ± 0.5, *n* = 28, *P* < 0.01 versus Ischemia) with a greater reduction of infarct volume, chiefly limited to the right striatum (4.5% ± 1.5%, *n* = 12, *P* < 0.01 versus Ischemia) (Table 1 and Figure 1(b)).

In the group of Ischemia + EA at left LI 11 and PC 6 (*n* = 24), 8% (2/24) of rats died within 2–10 hrs after MCAO (*P* < 0.01 versus Ischemia). At 24 h after reperfusion, the remaining rats showed significantly attenuated neurological deficits (Grade 2.0 ± 1.0, *n* = 22, *P* < 0.01 versus Ischemia) and a greatly reduced infarct volume (8.6% ± 3.8%, *n* = 12, *P* < 0.01 versus Ischemia) (Table 1 and Figure 1(c)). 

In the group of Ischemia + EA at right LI 11 and PC 6 (*n* = 24), 20% (5/24) of rats died within 2–20 hrs after MCAO (*P* > 0.05 versus Ischemia). At 24 h after reperfusion, the remaining rats showed serious neurological deficits (Grade 6.5 ± 0.5, *n* = 19, *P* > 0.05 versus Ischemia) with the cerebral infarct volume approximately one-third of the whole brain (33.4% ± 6.3%, *n* = 12, *P* > 0.05 versus Ischemia) (Table 1 and Figure 1(d)).

In the group of Ischemia + EA at left SP 6 and GB 34 (*n* = 24), 13% (3/24) rats died within 2–10 hrs after MCAO (*P* < 0.05 versus Ischemia). At 24 h after reperfusion, the remaining rats showed serious neurological deficits (Grade 6.0 ± 1.0, *n* = 21, *P* > 0.05 versus Ischemia) with the cerebral infarct volume approximately one-third of the whole brain (29.8%  ±  4.5%, *n* = 12, *P* > 0.05 versus Ischemia) (Table 1 and Figure 1(e)). 

### 3.2. EA-Induced Changes in Cerebral Blood Flow Varied with Different Acupoints

 Considering that an insufficient blood flow causes cerebral ischemic injury, we wondered if EA neuroprotection against cerebral ischemia was rendered through regulation of the blood supply to the ischemic brain and whether this regulation changed with a difference in the acupoints stimulated. Using a laser-doppler perfusion monitor, we monitored the real-time changes in cerebral blood flow in all experimental groups. 

**Figure 2:**
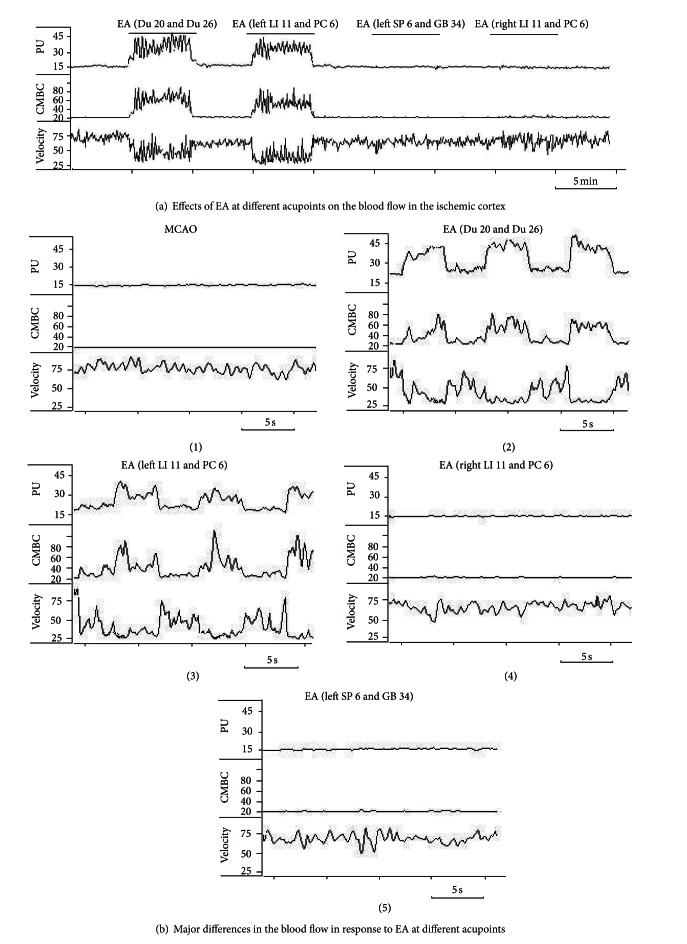

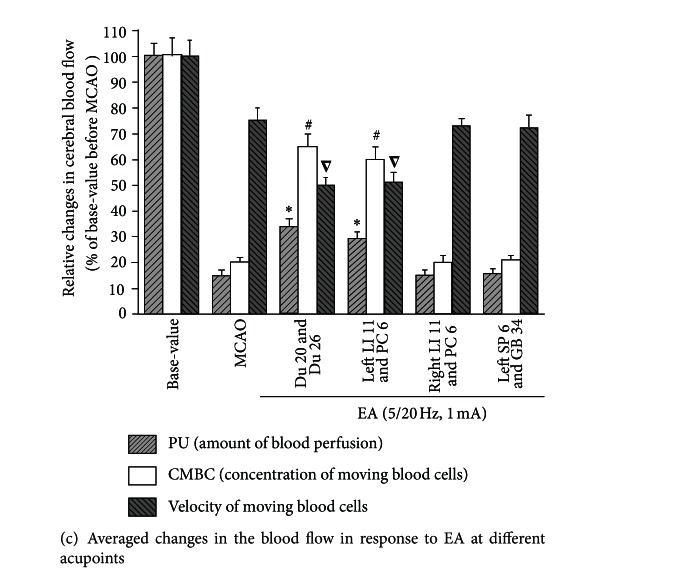
EA-induced changes in CBF in the ischemic cortex varied with stimulation of different acupoints. The CBF was measured in 6 ischemic rats subjected to EA treatment at different acupoints. Blood perfusion (PU), concentration of moving blood cells (CMBC), and velocity of blood cells (Velocity) were measured by a laser Doppler perfusion monitor system. (a) Changes in CBF in response to EA at different acupoints. (b) CBF in each EA condition. (c) Statistical summary of the changes in CBF in response to EA stimuli. *P < 0.01, MCAO versus MCAO plus EA (PU); ^#^ *P* < 0.01, MCAO versus MCAO plus EA (CMBC); ^∇^ *P* < 0.05, MCAO versus MCAO plus EA (Velocity). Note that EA at acupoints Du 20 and Du 26 and left LI 11 and PC 6 induced isochronous increase in PU and CMBC in addition to a decrease in Velocity, but EA at right LI 11 and PC 6 acupoints and left SP 6 and GB 34 acupoints had no significant effects on CBF despite using the same EA parameters.

Firstly, we tested if brief stimulation of these acupoints alters the blood flow on the ischemic rats (*n* ≥ 6, repeated ≥6 times for each pair of acupoints). EA with 5/20 Hz sparse-dense current at 1.0 mA was delivered to the acupoints in a manner of 5 min stimulation/5 min cessation. When a nylon suture was successfully inserted into the appropriate place of right middle cerebral artery, the blood perfusion of the monitored cortex decreased immediately from average 100 ± 20 PU to 15 ± 2 PU, that is, a ~85% drop in blood supply with a decrease in CMBC by ~80% and relatively slight deceleration of blood cell velocity by ~25% (*P* < 0.05) (Figures 2(a), 2(b)(1), and 2(c)). EA stimulation at Du 20 and Du 26 immediately induced a significant increase in blood flow. This increase in blood perfusion was synchronous to EA. Among the changes in blood flow, EA induced a 2-fold increase in PU (from ~15% to ~32% of the base level before MCAO, *P* < 0.01) with a 3-fold increase in CMBC (from ~20% to ~65% of the base level, *P* < 0.01) and a slight decrease in the Velocity (from ~75% to ~50% of the base-value, *P* < 0.05) (Figures 2(a), 2(b)(2), and 2(c)). Similarly, EA at left LI 11 and PC 6 also significantly increased the blood flow immediately after the onset of EA. During the EA stimulation, PU increased almost 2 folds (from ~15% to ~29% of the base-value, *P* < 0.01) with a significant 3-fold increase in CMBC (from ~20% to ~60% of the base level, *P* < 0.01) and a slight decrease in the Velocity (from ~75% to ~50% of the base level, *P* < 0.05) (Figures 2(a), 2(b)(3), and 2(c)). In sharp contrast, EA at right LI 11 and PC 6 (Figures 2(a), 2(b)(4), and 2(c)) and left SP 6 and GB 34 (Figures 2(a), 2(b)(5), and 2(c)) did not induce any significant changes in blood flow despite the use of same EA parameters.

**Figure 3:**
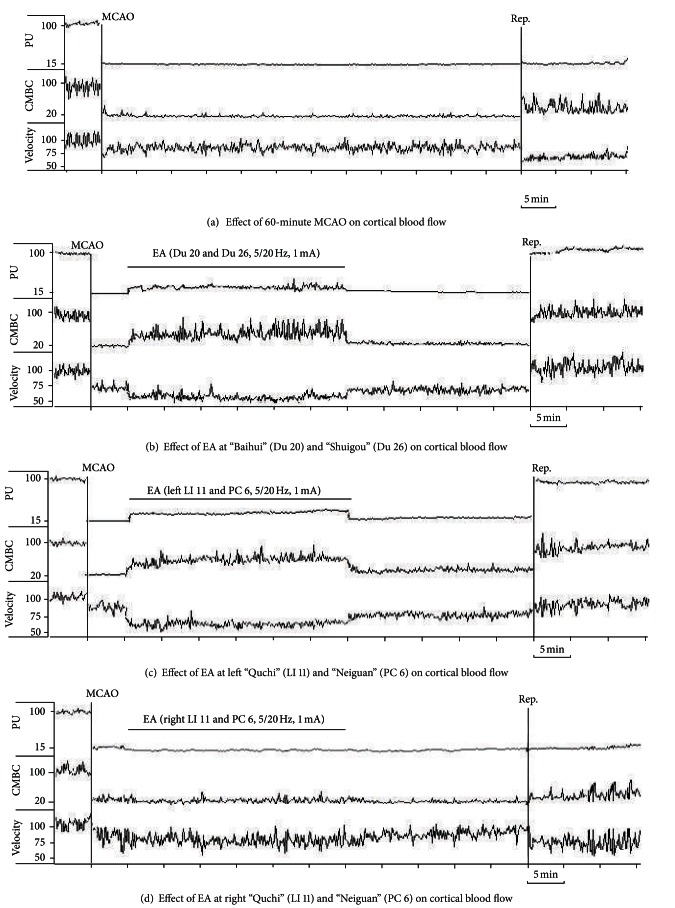

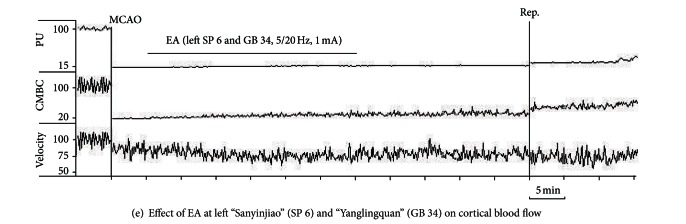
Representative trace recordings of the blood flow. Blood perfusion (PU), concentration of moving blood cells (CMBC), and Velocity of blood cells (Velocity) were measured in the ischemic rats by a laser Doppler perfusion monitor system. (a) Effect of MCAO-60 min on CBF during ischemia and reperfusion in the Ischemia group. (b) Effect of EA at acupoints Du 20 and Du 26 on CBF. (c) Effect of EA at left LI 11 and PC 6 on CBF. (d) Effect of EA at acupoints right LI 11 and PC 6 on CBF. (e) Effect of EA at acupoints left SP 6 and GB 34 on CBF. Note that the PU and CMBC decreased immediately after the right middle cerebral artery was occluded by a nylon suture. The blood flow was kept at a low level with fluctuant waves during the entire MCAO duration. After onset of reperfusion, PU and CMBC increased while the velocity further decreased. EA at right LI 11 and PC 6 acupoints induced no significant change in the CBF during or after MCAO. EA at left SP 6 and GB 34 acupoints induced no significant change in the CBF during the early stages of MCAO, but slightly increased the CMBC after a 10–15 min period of EA. EA stimulation at acupoints Du 20 and Du 26 or left LI 11 and PC 6 induced an isochronous increase in PU and CMBC with a decrease in velocity. After reperfusion, PU, CMBC, and Velocity all increased rapidly and reached the baseline values.

**Figure 4:**
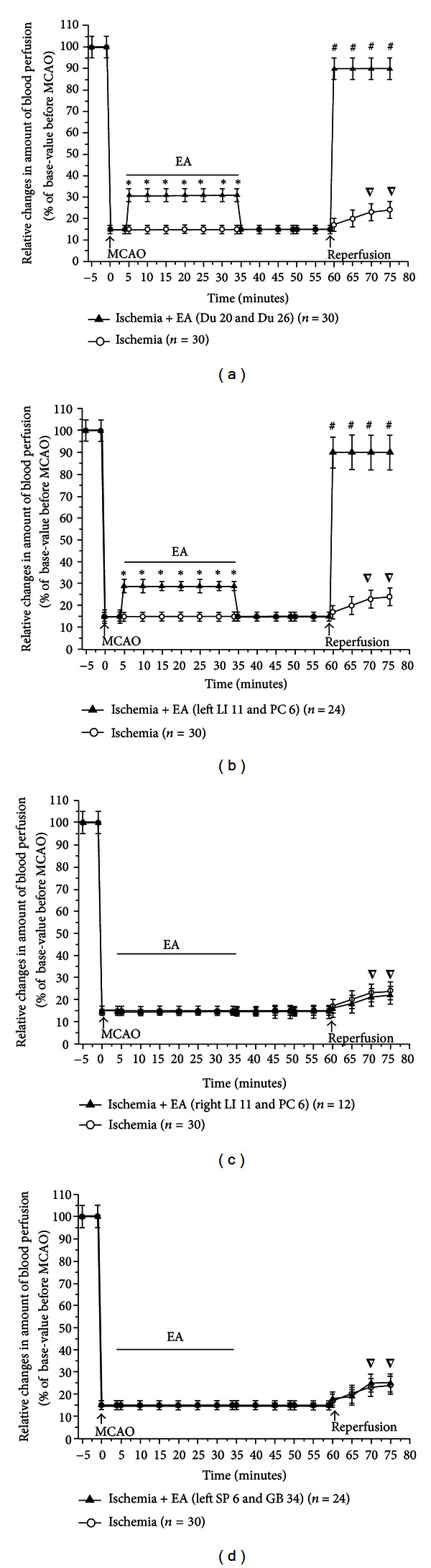
Statistical data on cerebral blood flow (PU) in ischemic cortex in MCAO/reperfusion rats with/without EA at different acupoints. PU was measured and compared between the groups. *P < 0.01, MCAO + EA versus MCAO; ^∇^ *P* < 0.05, reperfusion versus MCAO; ^#^ *P* < 0.01, reperfusion in MCAO + EA group versus reperfusion in MCAO group. Note that in the Ischemia group, MCAO (0 min) decreased PU to ~15% of the base level before MCAO (−5 min). After the onset of reperfusion, PU increased slightly to ~25%. In the group of EA at Du 20 and Du 26, EA increased PU to ~32% of base level before MCAO. In the group of EA at left LI 11 and PC 6, EA also increased PU to ~29% of base level before MCAO. After the onset of reperfusion, PU in the groups of EA at Du 20 and Du 26 and left LI 11 and PC 6 almost immediately returned to >90% of the pre-MCAO level. However, EA at right LI 11 and PC 6 and left SP 6 and GB 34 did not significantly improve the CBF during MCAO and after reperfusion.

**Figure 5:**
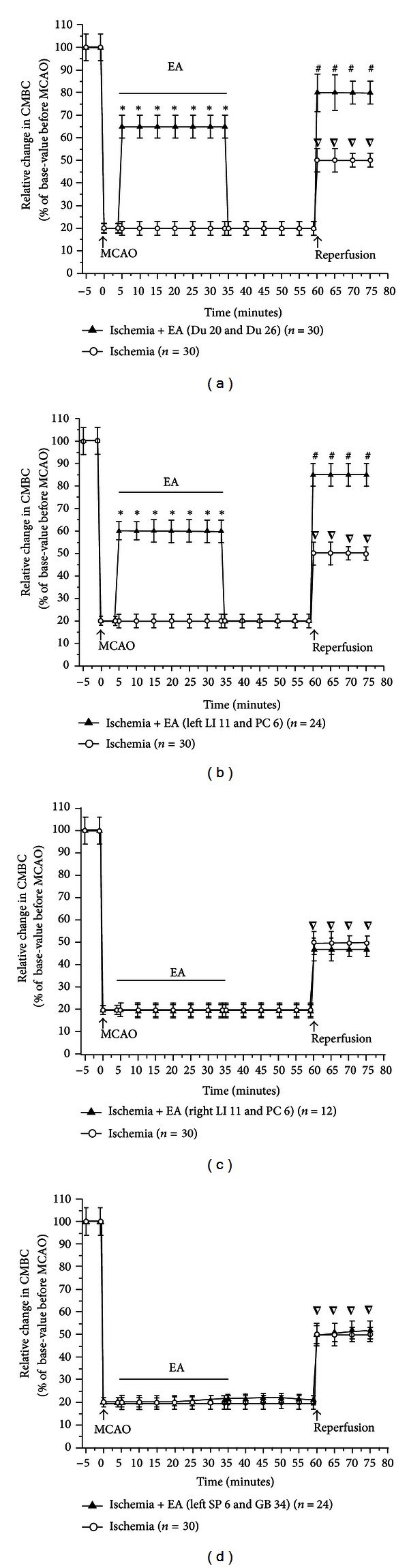
Statistical data on the concentration of moving blood cells (CMBC) in the ischemic cortex in MCAO/reperfusion rats with/without EA at different acupoints. CMBCs were measured and compared between the groups. *P < 0.01, MCAO + EA versus MCAO; ^∇^ *P* < 0.01, reperfusion versus MCAO; ^#^ *P* < 0.01, reperfusion in MCAO + EA group versus reperfusion in MCAO group. Note that in the Ischemia group, MCAO (0 min) decreased CMBC to ~20% of the base level before MCAO (−5 min). After the onset of reperfusion, CMBC increased to ~55% of the base level. In the group of EA at Du 20 and Du 26, EA increased CMBC to ~65% of the base level. In the group of EA at left LI 11 and PC 6, EA increased CMBC to ~60% of the base level. After the onset of reperfusion, CMBC in the groups of EA at Du 20 and Du 26 and left LI 11 and PC 6 recovered faster than that in the Ischemia group. However, EA at right LI 11 and PC 6 or at left SP 6 and GB 34 did not significantly improve the CMBC.

**Figure 6:**
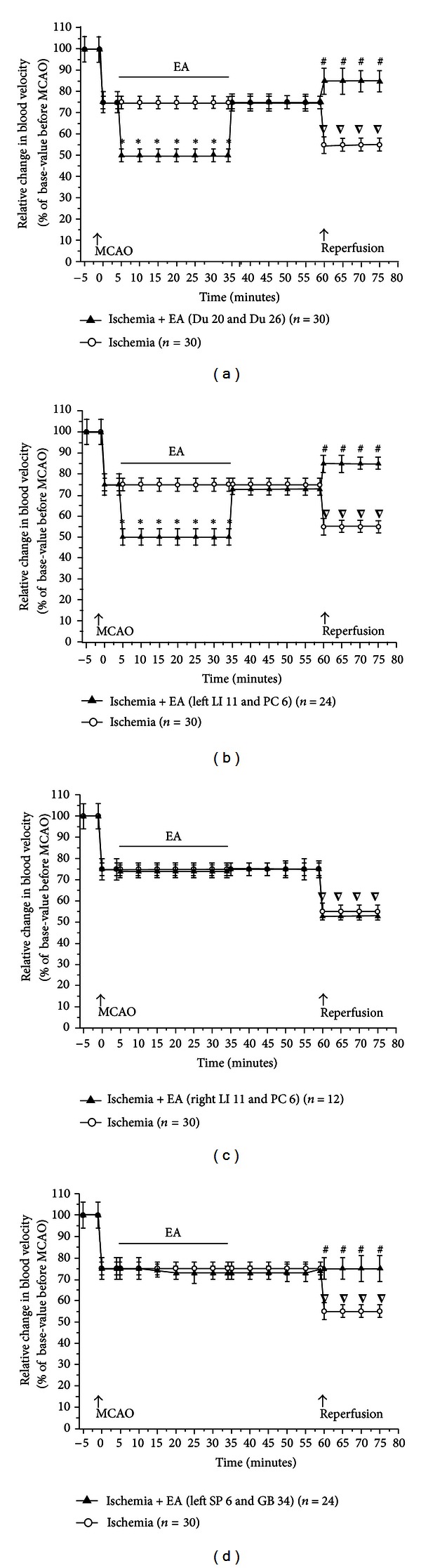
Statistical data representing the relative changes in velocity of the moving blood cells (Velocity) in the ischemic cortex in MCAO/reperfusion rats with/without EA at different acupoints. Velocities were measured and compared between the groups. *P < 0.05 MCAO + EA versus MCAO; ^∇^ *P* < 0.05 reperfusion versus MCAO; ^#^ *P* < 0.05, reperfusion in MCAO + EA group versus reperfusion in MCAO group. Note that in the Ischemia group, MCAO (0 min) decreased Velocity to ~75% of the base level before MCAO (−5 min). After the onset of reperfusion, Velocity decreased further to 50% of baseline (before MCAO) level. In the EA at Du 20 and Du 26 or left LI 11 and PC 6 groups, EA decreased the Velocity from 75% to 50% of the baseline (*P* < 0.05). After the onset of reperfusion, Velocity in the groups of EA at Du 20 and Du 26 and left LI 11 and PC 6 recovered faster than that in the Ischemia group (*P* < 0.05). However, EA at right LI 11 and PC 6 did not show a significant improvement in the velocity of moving blood cells. EA at left SP 6 and GB 34 acupoints showed a slight improvement in the velocity (*P* < 0.05).

 Based on these initial observations, we systematically investigated the changes in blood flow from the time before MCAO to the early stage of the reperfusion. Specifically, the recording started 5 mins prior to MCAO and continued on to 15 mins after the onset of reperfusion (suture withdrawal). EA started at 5 min after the onset of MCAO for a duration of 30 mins with the same stimulation parameters, that is, 5/20 Hz sparse-dense pulse at 1.0 mA in all groups (Figures 3–6). We then compared the differences in cerebral blood flow at the pre-MCAO level, and during MCAO with/without EA in various groups. 

 In the Ischemia group (MCAO for 60 min, *n* = 30), there was an immediate ~85% decrease in blood perfusion of the monitored cortex. The blood flow was kept constant at this low level for the entire duration of MCAO. The CMBC also decreased to ~20% of the baseline level (the level before MCAO), and the blood cell velocity decreased to 75% of the baseline (Figures 3(a) and 4–6).

In the group of Ischemia + EA at Du 20 and Du 26 (*n* = 30), EA significantly increased the blood flow immediately after the onset of stimulation with the increase in blood perfusion being synchronous to EA application. The EA-induced changes in blood flow induced a 2-fold increase in PU over the ischemic level (*P* < 0.01), CMBC increased by over 3 folds (*P* < 0.01), and the Velocity slightly decreased by ~33% (*P* < 0.05) (Figures 3(b), 4(a), 5(a), and 6(a)). As the stimulation ceased, the blood flow immediately decreased to the MCAO level. 

In the group of Ischemia + EA at left LI 11 and PC 6 (*n* = 24), EA significantly increased regional blood flow immediately after application. Specifically, PU increased by 2 folds over the ischemic (MACO) level (*P* < 0.01), CMBC significantly increased by 3 folds (*P* < 0.01), and the Velocity slightly decreased by >30% of the MCAO level (*P* < 0.05) (Figures 3(c), 4(b), 5(b), and 6(b)). As the stimulation ceased, the blood flow rapidly returned to the MCAO level.

In sharp contrast to the previous groups, EA at right LI 11 and PC 6 (*n* = 12) did not induce any significant changes in blood flow during MCAO (Figures 3(d), 4(c), 5(c), and 6(c)). The same was true for EA at left SP 6 and GB 34 (*n* = 24). In this group, EA did not significantly alter the blood flow in the first 10–15 minutes. In the later stage of EA, however, CMBC gradually increased with a slight reduction in the Velocity, although, the value of PU showed no significant change (Figures 3(e), 4(d), 5(d), and 6(d)).

### 3.3. Recovery of the Blood Flow during Reperfusion Varied among Various Groups

In the Ischemia group, following nylon suture removal after a 60-min MCAO, PU slightly increased to 25% of the control (pre-MCAO) level (*P* < 0.05 versus MCAO) and CMBC increased to ~55% of the control level (*P* < 0.01 versus MCAO). However, the velocity of blood cells further decreased during the transition from ischemia to reperfusion, that is, from 75% to 50% of the control (pre-MCAO) level (*P* < 0.05 versus MCAO) in the first 15 minutes after the blood reperfusion. These results indicate a constant hypoperfusion during MCAO and even after reperfusion, which was mainly due to a decrease in moving blood cells (CMBC) in MCAO or a reduction of blood cell velocity during reperfusion (Figures 3(a) and 4–6).

In the group of Ischemia + EA at Du 20 and Du 26 (Figures 3(b), 4(a), 5(a), and 6(a)) and the the group of Ischemia + EA at left LI 11 and PC 6 (Figures 3(c), 4(b), 5(b), and 6(b)), the cerebral blood flow (PU, CMBC, and Velocity) gradually increased to the control (pre-MCAO) during reperfusion. 

The group of Ischemia + EA at right LI 11 and PC 6 demonstrated a comparable change in the blood flow during reperfusion as seen in the Ischemia alone group (Figures 3(d), 4(c), 5(c), and 6(c)). The group of Ischemia + EA at left SP 6 and GB 34 (Figures 3(e), 4(d), 5(d), and 6(d)) showed changes in the PU and CMBC comparable to those of the Ischemia alone group during the beginning of reperfusion. However, the velocity recovered faster in this group as opposed to the ischemia alone group. In this EA group, the blood perfusion was largely improved starting from about 10–15 minutes after the onset of perfusion.

### 3.4. EA Had No Appreciable Effect on Cerebral Blood Flow in the Non-Ischemic Brain

**Figure 7:**
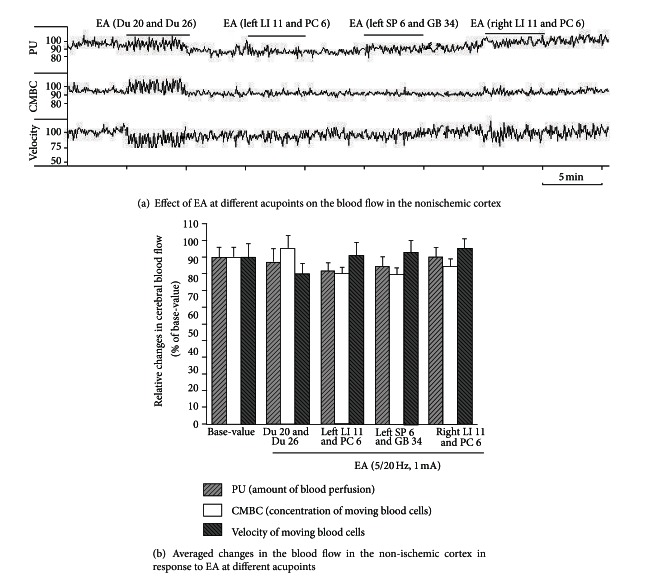
EA had no significant effect on the cerebral blood flow in the non-ischemic cortex at different acupoints. Blood perfusion (PU), concentration of moving blood cells (CMBC), and velocity of blood cells (Velocity) were measured in the non-ischemic rats by a laser Doppler perfusion monitor system. (a) Effects of EA at different acupoints on the CBF. (b) Statistical summary. Note that EA had no significant effect on PU, CMBC, and Velocity in response to EA stimulation at different acupoints in the non-ischemic cortex.

 Since EA at some acupoints, like Du 20 and Du 26 and left LI 11 and PC 6, significantly increased the blood flow to the ischemic brain, we further asked whether this response was specific to the ischemic brain or a generic brain response to EA stimulation. To investigate this, we applied EA at 1.0 mA with 5/20 Hz sparse-dense pulse to the same acupoints on the age-matched control rats and measured the changes in cerebral blood flow (*n* = 8). Interestingly, we found no significant effect of EA on cerebral blood flow in the non-ischemic animals despite using the same EA parameters used on the ischemic rats (Figure 7). These observations suggest that the application of EA at specific acupoints increased blood flow to the ischemic but not to the non-ischemic brain.

## 4. Discussion

This is the first study to systematically compare the effects of EA at different acupoints on cerebral blood flow and ischemic injury in the brain. Our comprehensive data specifically shows that EA at Du 20 and Du 26 induced maximum protection against cerebral ischemia as opposed to the limb acupoints. The stimulation of the contralateral (to the ischemic side) acupoints (LI 11 and PC 6) in the left forelimb also induces a significant cerebral protection against ischemic injury, while stimulation of the same acupoints in the ipsilateral forelimb and contralateral acupoints in the hindlimb (SP 6 and GB 34) had no such effect. 

In this work, we used optimized parameters, that is, 5/20 Hz parse-dense wave at 1.0 mA, for EA stimulation that confers optimal stimulation based on our previous systematic study [9, 13]. Since we used these parameters for all acupoints under the same experimental conditions, the differences in outcomes observed among all experimental groups can be attributed to specific effects of EA at these acupoints. Therefore, it is important to choose specific acupoints in the treatment of hypoxic/ischemic encephalopathy, such as stroke. Our observations may provide a useful resource for a standardized application of acupuncture in the treatment of stroke under clinical settings. 

Since the major cause of ischemic injury is insufficient blood supply to the brain, any change in blood flow may greatly affect the degree of brain injury. Our results show that in all “EA effective” groups, EA significantly increased the blood flow to the ischemic brain, thereby attenuating the effects of the hypoxic/ischemic environment in the brain. This further suggests the critical dependence of EA-induced protection on the improvement of cerebral blood flow. 

 The increase in the blood flow started soon after the onset of EA and disappeared almost immediately after the cessation of EA. Therefore, a quick and short-lasting mechanism underlies this action, quite possibly involving the nervous system. During EA stimulation, the muscles around the acupuncture needles showed slight contractions in response to the EA rhythm. It is possible that the currents directly stretch the tissue and physically affect the blood vessels, thus leading to an increase in the blood flow. However, the facts we noticed in this study do not support this possibility. For example, EA at right LI 11 and PC 6, the acupoints that were physically closer to the ischemic region (right side of the brain), did not increase the blood flow or protect the brain at all. In sharp contrast, EA at left LI 11 and PC 6, contralateral to the ischemic side and relatively farther away from the ischemic region, caused a significant increase in the blood flow and better protected the brain against ischemic injury. This observation provides further evidence in support of the generation of EA signals from peripheral nerves and their transmission to the contralateral brain to regulate the neurotransmitter release and neural function, which in turn regulates various bodily phenomena [19–22]. Altogether, the EA-induced increase in blood flow is most likely based on a neural-controlled regulation. However, the precise mechanisms of how the EA signal upregulates blood flow in the ischemic brain is unknown at this stage and requires further research.

Interestingly, we did not note any appreciable increase in the blood flow of the non-ischemic brain when the animals were stimulated with the same EA parameters. This suggests that the EA-induced regulation of the cerebral blood flow varies with the conditions of the organ/body. Under normal conditions, the EA signal may not change the blood flow to the brain since it is unnecessary. Under a stress like hypoxia/ischemia, however, the same EA signal grants the brain the ability to adapt to the hypoxic/ischemic microenvironment by increasing local blood perfusion. Therefore, this EA-mediated upregulation of cerebral blood flow can be a useful strategy not only for ischemic stroke, but also for cerebral vasospasm and other similar disorders. 

 In summary, our work evidently demonstrates the specificity of acupoints in EA protection against cerebral ischemia and suggests that acupoints in the head or contralateral forelimb are effective in the treatment of stroke. Stimulation of these acupoints by optimized EA parameters generates a signal to the brain that results in an increase in cerebral blood flow and rescues the brain from ischemic injury. Although the underlying mechanisms need further research, the EA-induced upregulation of cerebral blood flow is an effective strategy with a potential for clinical application in a variety of neurological conditions.

## Figures and Tables

**Table 1:** Effects of EA at different acupoints on cerebral ischemia.

Groups	Scales of neurological deficit	Infarct volume	Death rate
Ischemia	6.0 ± 0.5 (6 ~ 7) (*n* = 50)	32.9% ± 3.8% (*n* = 18)	17% (10 out of 60)
Ischemia + EA (Du 20 & Du 26)	1.0 ± 0.5 (0 ~ 2) (*n* = 28)^△^	4.5% ± 1.5% (*n* = 12)*	7% (2 out of 30)^¥^
Ischemia + EA (left LI 11 & PC 6)	2.0 ± 1.0 (1 ~ 3) (*n* = 22)^△^	8.6% ± 3.8% (*n* = 12)*	8% (2 out of 24)^¥^
Ischemia + EA (right LI 11 & PC 6)	6.5 ± 0.5 (6 ~ 7) (*n* = 19)	33.4% ± 6.3% (*n* = 12)	20% (5 out of 24)
Ischemia + EA (left SP 6 & GB 34)	6.0 ± 1.0 (5 ~ 7) (*n* = 21)	29.8% ± 4.5% (*n* = 12)	13% (3 out of 24)^#^

Ischemia: the rats were subjected to right MCAO for 1 hour and reperfusion for 24 hours. Ischemia + EA at Du 20 & Du 26: EA (1.0 mA, 5/20 Hz, sparse-density wave) was delivered to the acupoints of “Baihui” (Du 20) and “Shuigou” (Du 26) of the ischemic rats for 30 min. Ischemia + EA at left LI 11 & PC 6: EA was delivered to the acupoints of “Quchi” (LI 11) and “Neiguan” (PC 6) on the left anterior limb of the ischemic rats for 30 min. Ischemia + EA at right LI 11 & PC 6: EA was delivered to the acupoints of “Quchi” (LI 11) and “Neiguan” (PC 6) on the right anterior limb of the ischemic rats for 30 min. Ischemia + EA at left SP 6 & GB 34: EA was delivered to the acupoints of “Sanyinjiao” (SP 6) and “Yanglingquan” (GB 34) on the left posterior limb of the ischemic rats for 30 min.

^△^ *P* < 0.01 versus Ischemia. *P < 0.01 versus Ischemia. ^¥^ *P* < 0.01 versus Ischemia.^#^ *P* < 0.05 versus Ischemia. Note that both EA at Du 20 & Du 26 and EA at left LI 11 & PC 6 significantly reduced the neurological deficit, brain infarct volume, and death rate.
